# Treatment of acute pancreatitis with protease inhibitors administered through intravenous infusion: an updated systematic review and meta-analysis

**DOI:** 10.1186/1471-230X-14-102

**Published:** 2014-05-30

**Authors:** Takeshi Seta, Yoshinori Noguchi, Satoru Shikata, Takeo Nakayama

**Affiliations:** 1 Division of Gastroenterology and Hepatology, Japanese Red Cross Society Wakayama Medical Center, 4-20, Komatsubara-dori, Wakayama, Wakayama 640-8558, Japan; 2 Division of General Internal Medicine, Japanese Red Cross Society Nagoya Daini Red Cross Hospital, Myoken-cho 2-9, Showa-ku, Nagoya-city 466-8650, Aichi, Japan; 3 Department of Family medicine, Mie Prefectural Ichishi Hospital, 616 Minami ieshiro, Hakusan-cho, Tsu-city, Mie 515-3133, Japan; 4 Department of Health Informatics, Kyoto University School of Public Health, Yoshida-Konoe, Sakyo-ku, Kyoto, Kyoto 606-8501, Japan

**Keywords:** Acute pancreatitis, Protease inhibitors, Mortality, Control mortality rate, Complication

## Abstract

**Background:**

The intravenous use of protease inhibitors in patients with acute pancreatitis is still controversial. The purpose of this study was to evaluate the effectiveness of protease inhibitors intravenously administered to prevent pancreatitis-associated complications.

**Methods:**

We updated our previous meta-analysis with articles of randomized controlled trials published from January 1965 to March 2013 on the effectiveness of protease inhibitors for acute pancreatitis. A systematic search of PubMed, EMBASE, the Cochrane Library, and Japana Centra Revuo Medicina was conducted. In addition, Internet-based registries (ClinicalTrials.gov, controlled-trials.com, UMIN, JMACCT, and JAPIC) were used to search for on-going clinical trials. Furthermore, references of review articles and previously published meta-analyses were handsearched. The main outcome of interest was the overall mortality rate from acute pancreatitis.

**Results:**

Seventeen trials were selected for analysis. Overall, protease inhibitors did not achieve a significant risk reduction in mortality (pooled risk difference [RD], -0.02; 95% Confidence Interval [CI], -0.05 to 0.01; number needed to treat [NNT], 74.8) with low heterogeneity. A subgroup analysis in moderate to severe pancreatitis (defined by control mortality rate [CMR] >0.10) did not show a significant effect of protease inhibitors to prevent death (pooled RD, -0.03; 95% CI, -0.07 to 0.01; NNT, 1603.9) with low heterogeneity. An additional subgroup analysis of two trials with CMR >0.20 (i.e., low quality) revealed a significant risk reduction.

**Conclusion:**

The present meta-analysis re-confirmed that there is no solid evidence that supports the intravenous use of protease inhibitors to prevent death due to acute pancreatitis.

## Background

Acute pancreatitis is characterized by sudden abdominal pain and elevated serum concentrations of pancreatic enzymes. Overall mortality has been reported to be approximately 5 percent for acute pancreatitis and 20 percent for necrotizing pancreatitis [1,2]. Mild acute pancreatitis is generally treated with supportive care including pain control, intravenous fluids, and correction of electrolyte and metabolic abnormalities. A meta-analysis of eight trials revealed that enteral nutrition significantly reduced mortality, multiple organ failure, systemic infections, and the need for surgery compared with parenteral nutrition [3]. Furthermore, other meta-analyses that have been conducted on the effectiveness of acute pancreatitis treatments showed that the use of suitable analgesics effectively relieved pain [4], and that H_2_ receptor antagonists did not improve clinical outcomes of acute pancreatitis [5].

The role of protease inhibitors in the treatment of mild to severe acute pancreatitis is still unclear, although previous studies have demonstrated a marginal reduction in mortality. In particular, continuous regional arterial infusion of protease inhibitors was shown to be effective for acute necrotizing or severe pancreatitis in observational studies [6-8], as well as in a randomized controlled trial (RCT) [9]. In 2004, we conducted a meta-analysis of 10 RCTs with a total sample size of 1,036 [10] to evaluate the effectiveness of intravenous use of protease inhibitors for acute pancreatitis. The main outcome was the effectiveness of protease inhibitors to prevent death due to acute pancreatitis, and secondary outcomes were prevention of pancreatic pseudocyst, intra-abdominal abscess, and surgical intervention. Our analysis revealed no improvement in all outcomes. Moreover, protease inhibitors did not significantly reduce the mortality in the trials with control mortality rate (CMR) equal to or less than 0.10, but might reduce the mortality with CMR more than 0.10.

The purpose of the present study was to update the body of evidence on the effectiveness of intravenous use of protease inhibitors for acute pancreatitis.

## Methods

A systematic review of meta-analyses was conducted and the results were described according to the PRISMA statement [11].

### Literature search

First, we systematically searched PubMed, EMBASE, the Cochrane Library, and Japana Centra Revuo Medicina (the largest database of Japanese articles) for articles of RCTs published from January 1965 to March 2013 (search date: April, 2013) on the effectiveness of protease inhibitors used to treat acute pancreatitis. The electronic database search was conducted using a combination of Medical Subject Heading terms and text words “protease inhibitors” and “acute pancreatitis”. Next, we searched Internet-based clinical trial registries, ClinicalTrials.gov [12] and controlled-trials.com [13], as well as all the three trial registries available in Japan, UMIN [14], JMACCT [15], and JAPIC [16] (search date: April, 2013) for on-going RCTs using the same criteria. References of review articles and previously published meta-analyses were handsearched.

### Inclusion and exclusion criteria

Given that the purpose of the present study was to update the body of evidence regarding the effectiveness of intravenous use of protease inhibitors for acute pancreatitis, the following inclusion criteria were set: 1) randomized placebo-controlled trials of protease inhibitors administered through intravenous infusion; and 2) written in all languages. No restrictions were placed on severity of pancreatitis or type of protease inhibitors. Furthermore, we excluded trials in which 1) both intervention and control groups were administered protease inhibitors; 2) protease inhibitors were administered by intra-arterial or intra-abdominal infusion; 3) subjects included patients with chronic pancreatitis; 4) pancreatitis was noted after endoscopic retrograde cholangiopancreatography (ERCP); 5) patients were administered frozen plasma; 6) the goal was to investigate basic medical science (e.g., pancreatic enzyme research); 7) patients had human immunodeficiency virus; 8) efficacy of antibiotics was being evaluated; 9) patients included post-operative cases; and 10) oral administration of protease inhibitors.

No restrictions were placed on patient age, sex, or cause of acute pancreatitis. Articles in the form of a conference proceeding or full paper were also included. One author (TS) selected articles to be included for analysis, and the other authors verified the process.

### Outcome measures

The effectiveness of protease inhibitors for acute pancreatitis was evaluated based on the primary outcome of death due to acute pancreatitis, and secondary outcomes including relief from pain, pseudocyst formation, formation of intra-abdominal abscess, surgical intervention, paralytic small bowel obstruction, and other major complications including multiple organ failure.

### Study characteristics

Study design, participants, mode of intervention, and definition of outcomes were faithfully extracted from the articles included in the final analysis. We also confirmed the status of industrial support for each trial.

### Control mortality rate (CMR)

We calculated CMR (defined as the number of deaths in the control group divided by the number of patients in the control group) for each RCT.

### Quality assessment for selected trials and data extraction

The quality of each trial retrieved was assessed by the Jadad method [17] on the basis of whether the trial was randomized, the appropriateness of the randomization process (if applicable), whether the trial was double-blinded, the appropriateness of the double-blinding process (if applicable), and withdrawals/dropouts. Each item was assigned a score of 0 or 1, and the total score ranged from 0 to 5. In the present meta-analysis, trials with a Jadad score greater than 3 were defined as high-quality trials. Furthermore, we evaluated risk of bias for each trial and assessed the quality of the body of evidence using the Grading of Recommendations Assessment, Development, and Evaluation (GRADE) system for grading evidence [18].

One author (TS) conducted the quality assessment and extraction of analyzable data, which were verified by the other authors. Disagreement or uncertainty was resolved among all the authors.

### Statistical analysis

We calculated the risk difference (RD), i.e., risk in the intervention group *minus* risk in the control group, for the primary outcome of the trials. A negative RD indicated risk reduction due to intervention, and a positive RD, risk increase due to intervention (range, -1 to 1). Whether the treatment or control was favored was denoted by the signs “+” and “-”, respectively. Then, the weighted pooled estimates were calculated for binary data. A fixed-effect model weighted by the Mantel-Haenszel (M-H) method was used for pooling RD [19], followed by a test of homogeneity. Homogeneity among trials was assessed using the I^2^ test [20]. We defined I^2^ value <25% as low, 25 to 50% as moderate, and >50% as high heterogeneity.

If the hypothesis of homogeneity was rejected, a random-effect model using the DerSimonian-Laird (D-L) method was employed [21]. The potential for publication bias was examined by the funnel plot method [22] using the Begg’s [23] or Egger’s test [24]. The number needed to treat (NNT, 1/RD) to prevent one adverse event was also used as a measure of treatment effect. We used the “number needed to treat benefit (NNTB; the number of patients needed to be treated for one additional patient to benefit)” for a positive NNT, and the “number needed to treat harm (NNTH; the number of patients needed to be treated for one additional patient to be harmed)” for a negative NNT. When the upper or lower limit of the 95% confidence interval (CI) was infinity, the NNT scale including infinity was used [25]. All statistical analyses were performed with Stata statistical software [26]. Results were expressed as means and 95% CIs, unless otherwise indicated. P < 0.05 was considered statistically significant.

## Results

### Trial selection and features (Figure 1 and Additional file 1: Table S1)

**Figure 1:**
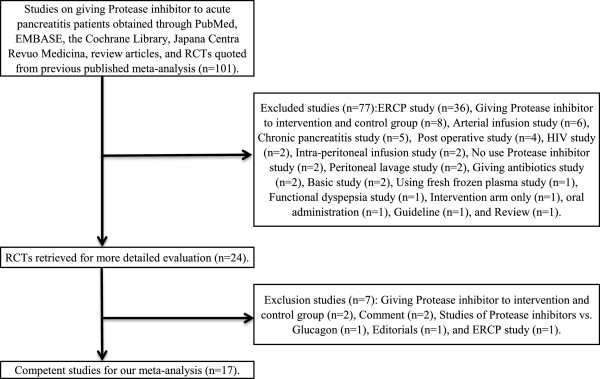
**Flow of randomized controlled trials through the process of retrieval and inclusion in the meta-analysis comparing protease inhibitors with placebo for acute pancreatitis.** The numbers in parentheses are the ‘Jadad scores’ of the individual trials. 95% CI, 95% confidence interval. ERCP, endoscopic retrograde cholangiopancreatography.

Our database search yielded 96 articles, and handsearching of bibliographies of retrieved meta-analyses and clinical guidelines yielded additional three and two articles, respectively. There were no on-going trials in the registries. Of the 101 articles, 24 met the inclusion criteria [27-50]; no multiple publications were found. Reviewers’ selection of relevant articles was completely the same, and there was no unsuitable study for inclusion by authors’ consensus. The 77 excluded articles described ERCP studies (n = 36), studies in which a protease inhibitor was administered to both intervention and control groups (n = 8), arterial infusion studies (n = 6), and other (n = 27) (Figure 1). Next, all authors read the selected 24 articles, reaching a consensus to exclude seven more articles [27,38-40,45,48,50] including two in which a protease inhibitor was administered to both intervention and control groups [30,45], two that were published as comments [38,50], one in which glucagon was given to the control group [39], one published as an editorial (n = 1) [40], and one reporting an ERCP study [48]. In the end, a total of 17 articles [27-29,31-37,41-44,46,47,49] were selected for analysis.

The present meta-analysis of the retrieved competent studies included 15 RCTs from the handsearch [27,28,31-37,41-43,46,47,49], one [29] from a previous meta-analysis [51], and one [44] from guidelines [52], with the total sample size of 1,697 patients. Of the 15 articles manually searched, 10 [33,34,37,41-44,46,47,49] were used in our previous meta-analysis [10].

All articles evaluated death due to acute pancreatitis, in addition to other outcomes such as pain relief (n = 2) [31,49], pseudocyst formation (n = 5) [29,37,41,43,46], intra-abdominal abscess formation (n = 4) [37,41,43,46], surgical intervention (n = 3) [44,47,49], paralytic small bowel obstruction (n = 3) [41,47,49], and other complications (n = 5) [36,37,43,46,47]. Of the 17 articles, 11 [29-31,33-36,41,42,44,46,47] were conducted in a multicenter setting, and six [27,28,32,37,42,49] in a single-center setting. Sixteen articles were published as a full paper [27-29,31-37,41-43,46,47], and one as an abstract only [44]. Fourteen articles were written in English [27-29,31,33-35,37,41,43,44,46,47,49], two in German [32,42], and one in French [36]. Eleven trials used aprotinin [27-29,31-37,41], and six trials used gabexate mesilate [42-44,46,47,49], all against the placebo control. Four articles [36,41,47,49] described tangible outcomes in the method section, whereas 13 provided no description [27-29,31-35,37,42-44,46]. The sample size was pre-calculated only in one article [47]. Acute pancreatitis was defined as clinical manifestation with elevated serum pancreatic enzyme levels in 11 articles [27-29,34,35,37,41,43,46,47,49], or as clinical manifestation with elevated urine pancreatic enzyme levels in two [32,33]; no description regarding the definition was found in the remaining four articles [31,36,42,44]. Nine articles described sources of funding or medicine support from industries [27,29,31,34,35,37,41-44]. The severity of acute pancreatitis was reported using the Ranson’s score in two articles [47,49], or Acute Pathophysiology and Chronic Health Evaluation (APACHE-II) score in one article [49]. In addition to these, we evaluated the severity of acute pancreatitis according to CMR in the present analysis, and found that 10 articles had CMR >0.10 [27,28,34-36,41,43,44,47,49]. No articles mentioned the presence or absence of adverse events. Seven articles observed death due to acute pancreatitis as the primary outcome [27,28,32-35,47]. Six articles reported no secondary outcome [27,28,32-35], while one article observed surgical intervention and bowel obstruction as the secondary outcomes [47]. Ten articles reported undistinguishable outcomes [29,31,36,37,41-45,49]. We performed quality of evidence assessment and found death, pseudocyst, intra-abdominal abscess, and any major complications to be low quality, and abdominal pain, surgical intervention, and bowel obstruction to be very low quality. Table 1 shows heterogeneity of each outcome with I^2^ values.

**Table 1:** Evaluation of evidence quality of using each RCT

**Author**	**Year**	**Allocation**	**Concealment**	**Blinding**	**Outcome data addressed**	**Selective outcome reporting**	**Other bias**	**ITT analysis**
Skyring A [27]	1965	no	yes	yes	no	no	no	no
Ryall RJ [28]	1966	no	no	no	no	no	no	no
Trapnell JE [29]	1967	no	yes	yes	no	no	no	no
Bachrach WH [31]	1968	no	yes	yes	no	no	no	no
Möller C [32]	1969	no	no	no	no	no	no	no
Baden H [33]	1969	no	yes	yes	no	no	no	no
Trapnell JE [34]	1974	no	yes	yes	no	no	no	no
MRC Multicenter Trial [35]	1977	no	yes	yes	no	no	no	no
Gauthier A [36]	1978	no	yes	yes	no	yes	no	no
Imrie CW [37]	1978	no	yes	yes	no	no	no	no
MRC Multicenter Trial [41]	1980	no	yes	yes	no	yes	no	no
Freise J [42]	1986	no	yes	yes	no	no	no	no
Yang CY [43]	1987	no	no	no	no	no	no	no
Goebell H [44]	1988	no	yes	yes	yes	no	no	no
Valderrama R [46]	1992	yes	yes	yes	yes	no	no	no
Büchler M [47]	1993	yes	yes	yes	yes	yes	no	yes
Chen HM [49]	2000	no	no	no	no	yes	no	no

*RCT* randomozed controlled trial; *ITT* intension to treat.

### Quality assessment (Additional file 1: Table S1 and Table 2)

**Table 2:** Effectiveness for acute pancreatitis with protease inhibitors

**Outcomes**	**No. of studies**	**Pooled risk difference**					**NNT**	**Heterogeneity**	**Statistical method by effect model**	**Quality of a body of evidence**
		**Value**	**95%CI**		**Value**		**95%CI**	**I**^**2**^**value(%)**		
			**Lower**	**Upper**	**NNTB**	**NNTH**				
Death	17	-0.02	-0.05	0.01	74.8		NNTH 62.4 to ∞ to NNTB 23.4	0	M-H	low
High quality studies	6	-0.02	-0.06	0.02		624.6	NNTH 24.6 to ∞ to NNTB 26.7	34.1	M-H	
Aprotinin	11	-0.01	-0.05	0.02	89.3		NNTH 38.8 to ∞ to NNTB 20.8	0	M-H	
Gabexate mesilate	6	-0.02	-0.07	0.03	54.3		NNTH 33.3 to ∞ to NNTB 14.9	31.1	M-H	
Daily dosage >900 mg of GM	5	-0.02	-0.09	0.04	55.2		NNTH 8.3 to ∞ to NNTB 14.C	47.0	M-H	
Daily dosage >1500 mg of GM	2	-0.09	-0.33	0.15	33.6		NNTH 17.8 to ∞ to NNTB 8.6	79.0	D-L	
Mild pancreatitis	7	0.00	-0.03	0.04		177.5	NNTH 24.5 to ∞ to NNTB 33.9	0	M-H	
Moderate to severe pancreatitis	10	-0.03	-0.07	0.01		1604	NNTH 23.0 to ∞ to NNTB 23.7	19.7	M-H	
Severe pancreatitis	2	-0.19	-031	-0.08	5.2		3.2 to 12.7	0	M-H	
With sponsor	9	-0.02	-0.06	0.02	67.0		NNTH 43.0 to ∞ to NNTB 18.8	15.0	M-H	
Without sponsor	8	-0.02	-0.06	0.03	68.4		NNTH 31.9 to ∞ to NNTB 16.5	0	M-H	
Abdominal pain	2	-0.26	-0.40	-0.13	3.9	2.5 to 9.6	85.0	D-L		very low
High quality study	1	-0.14	-0.32	0.03	6.9		NNTH 30.1 to ∞ to NNTB 3.1	Uncalculatable	M-H	
Pseudocyst formation	5	-0.00	-0.05	0.03	298.6		NNTH 27.3 to ∞ to NNTB 23.1	0	M-H	low
Intra-abdominal abscess formation	4	-0.01	-0.04	0.02	113.2		NNTH 65.3 to ∞ to NNTB 30.3	0	M-H	low
Surgical intervention	3	-0.08	-0.17	-0.00	11.8		6.0 to 443.8	60.5	D0L	very low
High quality study	1	0.00	-0.10	0.11		225.8	NNTH 8.9 to ∞ to NNTB 9.7	Uncalculatable	M-H	
Bowel obstruction	3	-0.06	-0.12	-0.00	6.3		4.0 to 14.5	58.8	D-L	very low
High quality study	1	-0.03	-0.08	0.02	33.9		NNTH 44.2 to ∞ to NNTB 12.3	Uncalculatable	M-H	
Any major complications	5	-0.01	-0.08	0.06	76.4		NNTH 15.7 to ∞ to NNTB 11.1	0	M-H	low

*NNT* number needed to treat; *NNTB* number needed to treat benefit; *NNTH* number needed to treat harm; *CI* confidence intervals; *M-H* mantel-haenszel; *D-L* dersimonian-laird; *GM*, gabexate mesilate.

The overall mean Jadad score was 2.1 (range 0–5). The mean Jadad score increased to 2.6 if three RCTs with a 0 score [28,32,43] were excluded. Six trials [31,34,35,37,46,47] were considered high quality (Jadad score ≥3), with the mean Jadad score of 3.7. With respect to risk of bias of each trial, two described random allocation [46,47], 13 described allocation concealment [27,29,31,33-37,41,42,44,46,47], 13 described blinding [27,29,31,33-37,41,42,44,46,47], three described outcome data addressed [44,46,47], and four described selective outcome reporting [36,41,47,49]; no other bias was described. One RCT described intention to treat (ITT) analysis [47]. The quality of each outcome in terms of evidence was graded low to very low.

### Primary outcome: preventing death (Tables 1 and 3)

**Table 3:** Subgroup analyses on the primary outcome (death due to acute pancreatitis)

**No**	**Subgroup**	**Trial (n)**	**Citations**	**Effectiveness**	**Heterogeneity**
1	High-quality	6	[31,34,35,37,46,47]	No significant	Moderate
2	Aprotinin	11	[27-29,31-37,41]	No significant	Low
3	Gabexate mesilate	6	[42-44,46,47,49]	No significant	Low to moderate
4	Gabexate mesilate daily administrated dosage>900mg	5	[42,44,46,47,49]	No significant	Low to moderate
5	Gabexate mesilate daily administrated dosage>1500mg	2	[47,49]	No significant	Low to moderate
6	Trials with CMR≦0.10	7	[29,31-33,37,42,46]	No significant	Low to moderate
7	Trials with CMR>0.10	10	[27,28,34-36,41,43,46,47,49]	No significant	Low to moderate
8	Trials with CMR>0.20	2	[34,49]	No significant	Low to moderate
9	Trials with industrial support	9	[27,29,31,34,35,37,41-43]	No significant	Low to moderate
10	Trials with industrial support	8	[28,32,33,36,44,46]	No significant	Low to moderate

High-quality was defined as Jadad score>3 points; *CMR* control mortality rate.

**Figure 2:**
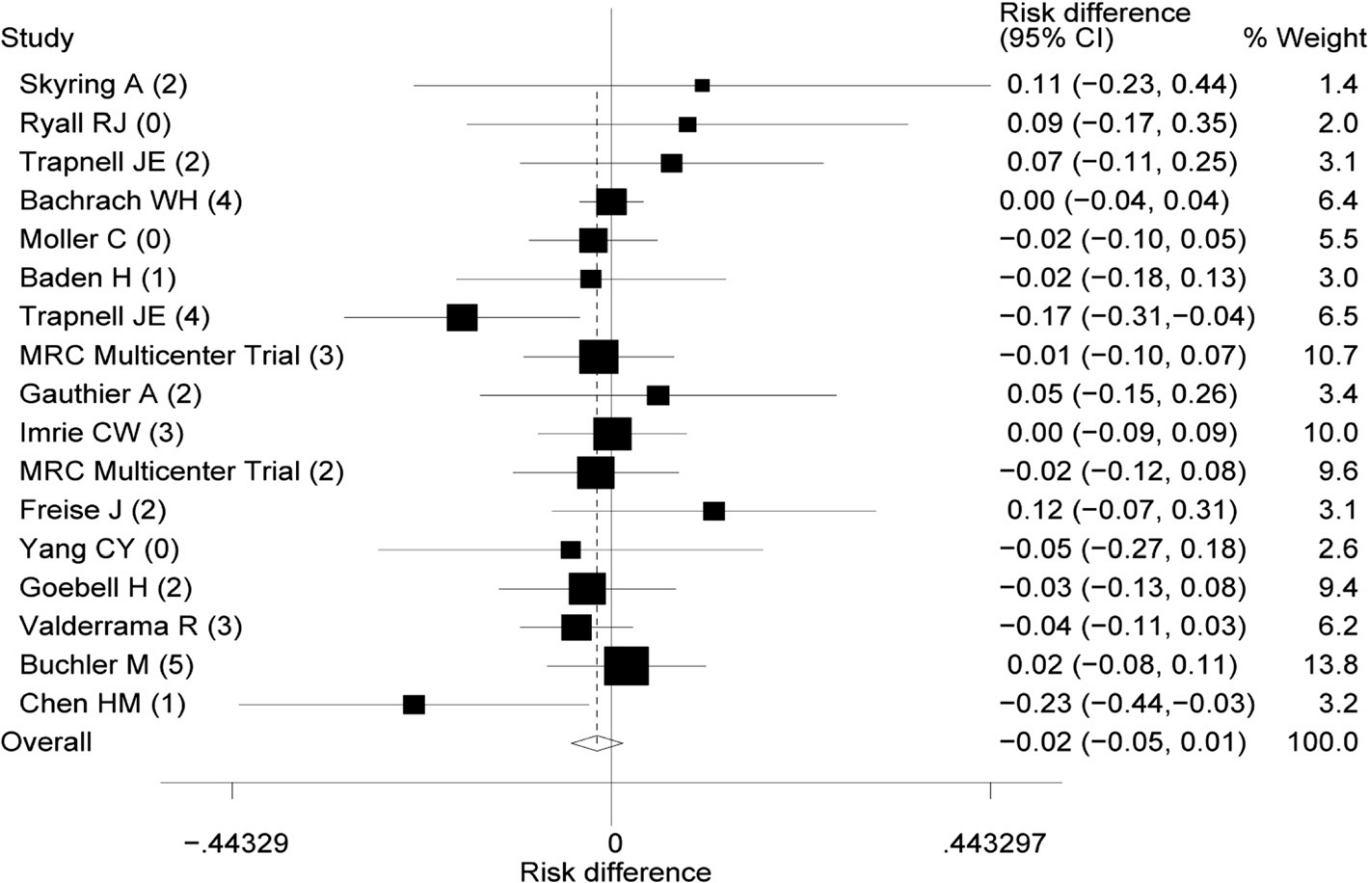
**Pooled risk difference (RD) in acute pancreatitis in patients given protease inhibitors.** The numbers in Parentheses are the “Jadad scores” of the individual trait. I^2^ value shown heterogeneity was 0%. 95% CI, 95% confidence interval.

**Figure 3:**
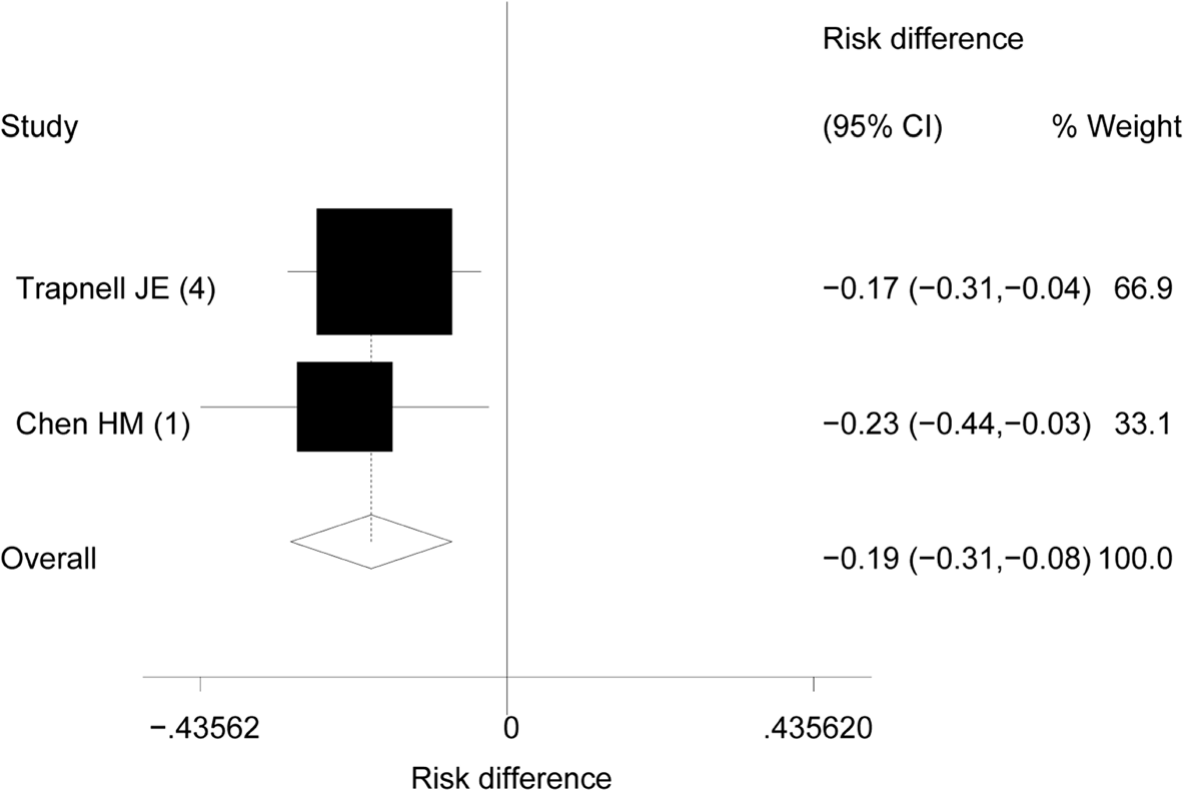
**Pooled risk difference (RD) in acute severe pancreatitis in patients given protease inhibitors.** The numbers in Parentheses are the “Jadad scores” of the individual trait. I^2^ value shown heterogeneity was 0%. 95% CI, 95% confidence interval.

All trials reported death due to acute pancreatitis (Table 1 and Figures 2 and 3), 11 with aprotinin [27-29,31-37,41], and six with gabexate mesilate [42-44,46,47,49]. Overall results of the 17 trials showed no significant risk reduction (low heterogeneity) in mortality with the use of protease inhibitors. Furthermore, subgroup analyses revealed no significant results (Table 3).

### Secondary outcomes

#### *Preventing abdominal pain*

Two trials reported that protease inhibitors (aprotinin [31] and gabexate mesilate [49]) were effective in preventing abdominal pain [31,49]. Overall results showed a significant risk reduction (high heterogeneity) with protease inhibitor use. However, the subgroup analysis of one of the two trials deemed high quality [31] revealed no significant effectiveness.

#### *Preventing pseudocyst formation*

Five trials (three for aprotinin [29,37,41] and two for gabexate mesilate [43,46]) reported that protease inhibitors were effective in preventing pseudocyst formation. Overall results showed no significant risk reduction (low heterogeneity).

#### *Preventing intra-abdominal abscess formation*

Four trials (two for aprotinin [37,41] and two for gabexate mesilate [43,46]) reported that protease inhibitors were effective in preventing intra-abdominal abscess formation. Overall results showed no significant risk reduction (low heterogeneity).

#### *Preventing surgical intervention*

Three trials, all of which evaluated the use of gabexate mesilate, reported that the protease inhibitor was effective in preventing surgical intervention [44,47,49]. Overall results showed a significant risk reduction (moderate to high heterogeneity). However, the subgroup analysis of one of the three trials deemed high quality [47] revealed no significant effectiveness.

#### *Preventing bowel obstruction*

Three trials (one for aprotinin [41] and two for gabexate mesilate [47,49]) reported that protease inhibitors were effective in preventing bowel obstruction. Overall results showed a significant risk reduction (moderate to high heterogeneity). However, the subgroup analysis of one of the three trials deemed high quality [47] revealed no significant effectiveness.

#### *Preventing major complications*

Five trials (two for aprotinin [36,37] and three for gabexate mesilate [43,46,47]) reported that protease inhibitors were effective in preventing some complications. These complications included respiratory failure, renal failure, gastrointestinal bleeding, metabolic failure (details unknown), sepsis, or hypoxia [43,46]. Three studies defined complications as pancreatitis-related complications or unknown [33,37,47]. Overall results showed no significant risk reduction (low heterogeneity).

### Publication bias (Figure 4)

**Figure 4:**
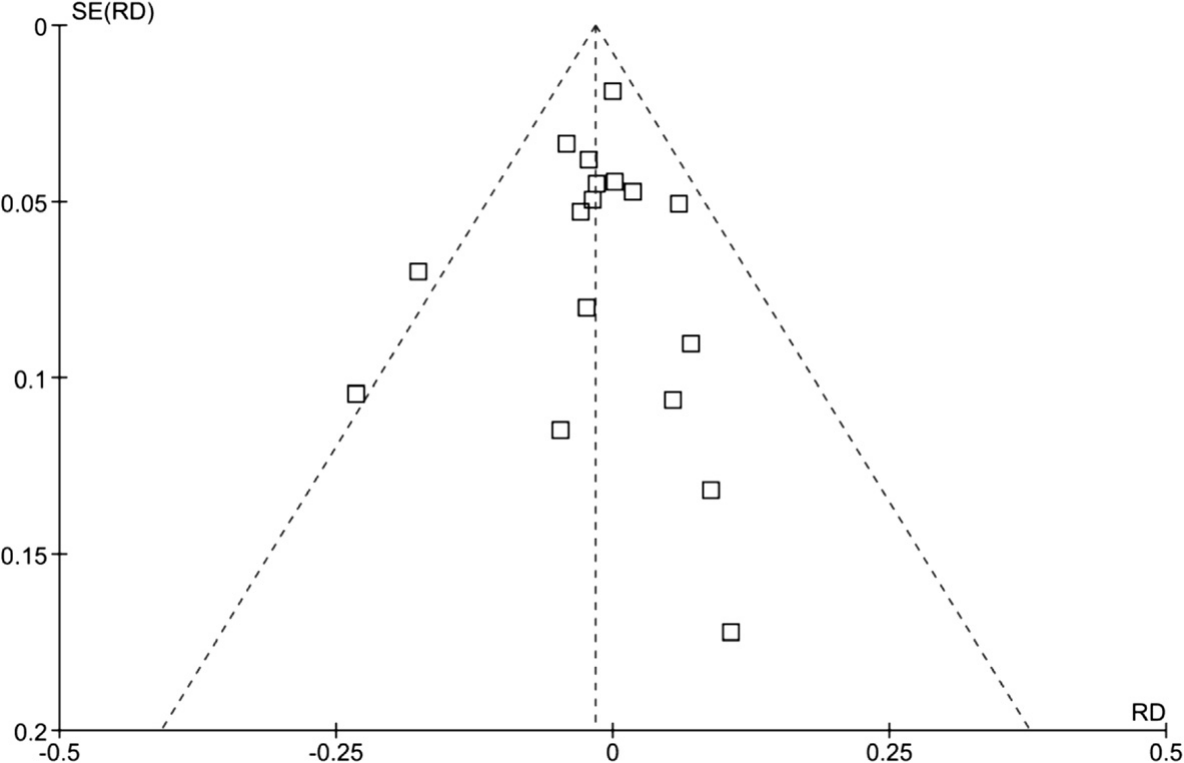
**Publication bias of trials reporting acute pancreatitis in patients given protease inhibitors.** RD, Risk Difference; SE, Standard Error.

The funnel plot was not symmetric; however, neither one of the statistical tests revealed significant publication bias (p = 0.653 and p = 0.736, respectively).

### Sensitivity analysis for trial quality (Tables 4 and 5)

**Table 4:** Sensitivity analysis for trial quality

	**No. of studies**	**Pooled RD**	**95% CI**
Overall	17	-0.02	-0.05 to 0.01
Jadad score ≧1	14	-0.02	-0.05 to 0.01
Jadad score ≧2	12	-0.01	-0.04 to 0.02
Jadad score ≧3	6	-0.02	-0.06 to 0.02

*RD* risk difference; *CI* confidence interval.

**Table 5:** Sensitivity analysis for control mortality rate

	**No. of studies**	**Pooled RD**	**95% CI**
Overall	17	-0.02	-0.05 to 0.01
CMR ≧0.10	10	-0.03	-0.07 to 0.01
CMR ≧0.20	2	-0.19	-0.31 to -0.08

*RD* risk difference; *CI* confidence interval; *CMR* control mortality rate.

Using the main outcome, i.e., overall mortality from acute pancreatitis, we performed a sensitivity analysis according to the Jadad score. The effect size and level of statistical significance did not decrease with increasing trial quality. We then performed another sensitivity analysis according to CMR. In all trials and trials with CMR <0.20, no significant effectiveness of protease inhibitors was shown. However, a significant effectiveness was found when trials were limited to those with CMR >0.20.

## Discussion

In this study, we conducted a systematic review of RCTs to update our previous meta-analysis of 2004 on the effectiveness of intravenous infusion of protease inhibitors for acute pancreatitis. As the present literature search retrieved no additional RCTs since 2004 up to March 2013, we analyzed a new set of articles including those that had not been identified in the previous meta-analysis [10]. The present analysis re-confirmed the validity of evidence reported to date, as follows: 1) treatment with protease inhibitors did not significantly reduce the mortality rate from acute pancreatitis; 2) subgroup analysis of trials with CMR greater than 0.20 showed limited effectiveness of protease inhibitors in preventing death; 3) protease inhibitors showed no significant effectiveness even in outcomes other than preventing death. Moreover, given that there are no on-going trials regarding the use of protease inhibitors for acute pancreatitis, no new evidence supporting their effectiveness is expected to emerge for the next few years.

In the previous meta-analysis, we evaluated prevention of death due to acute pancreatitis, formation of pancreatic pseudocysts, intra-abdominal abscess, and surgical intervention as outcomes. In the present analysis, we added prevention of abdominal pain due to acute pancreatitis and bowel obstruction. The present analysis had a larger sample size of 1,697 in total, compared to 1,036 in the previous analysis. The effectiveness of protease inhibitors stratified by CMR level was similarly examined in both the previous and present analyses. In the previous analysis [10], five [34,43,44,47,49] of 10 selected articles [31,33,34,37,42-44,46,47,49] were with CMR >0.10 and showed limited effectiveness. In the present analysis, 10 [27,28,34-36,41,43,44,47,49] of 17 selected articles were with CMR >0.10 and showed no significant effectiveness. On the other hand, the subgroup analysis of articles with CMR >0.20 [34,49] revealed a significant therapeutic effect. However, it is suspected that low-quality trials that included potentially severe patients might have exaggerated the effectiveness. In fact, the mean Jadad score was 2.5 in RCTs with CMR >0.20, and 1 point in those with CMR >0.30, suggesting that it is very likely that low-quality trials provided a veneer of effectiveness for severe patients.

The quality of evidence was assessed for each trial by two methods, one that used the conventional Jadad scoring system, and the other based on the GRADE system. The mean Jadad score (2.1 points) was low, and the majority of RCTs were deemed low to moderate quality, except for six trials [31,34,35,37,46,47] that were deemed high quality with a Jadad score of ≥3. We then used the GRADE system to evaluate each RCT in terms of risk of bias (random allocation, allocation concealment, blinding, outcome data addressed, selective outcome reporting, and others). Furthermore, we graded the quality of the body of evidence for each clinical outcome using the GRADE system in addition to the Jadad scoring system to further strengthen quality of evidence assessment. Handling of outcome data was likely to become a nest of bias, reporting bias, and ITT analysis were mentioned in three articles [44,46,47], four articles [36,41,47,49], and one article [47], respectively. The quality of RCTs included in the present review was low in general, and hence, the bodies of evidence and result estimations are both likely to be low in quality. We also checked each article in terms of whether there were descriptions regarding industrial support, tangible outcome, and sample size calculation in the methodology. With respect to industrial support, we found no significant difference in effectiveness according to the status of industrial support; however, eight articles [28,32,33,36,44,46,47,49] provided no description regarding funding or medicine support, and thus the actual status was unknown. With respect to tangible outcomes, only four of 17 articles [36,41,47,49] provided descriptions in the methods sections, and no article reported the presence or absence of adverse events. Sample size calculation was described in only one article [47]. The CONSORT statement [51], which specifies reporting of the primary outcome and sample size calculation, might have led to improved quality of reporting [52,53], although previous studies found inappropriateness or deficit of reporting common in published articles [54,55]. It was likely for the authors of adopted trials to select only the preferable results to report, among many outcomes measured.

This study has some limitations worth noting. First, the subgroup analysis which aimed to consider disease severity was not based on established criteria of severity. Instead, we defined CMR >0.10 as an arbitrary and retrospective index to indicate moderate to severe pancreatitis. Prospective indices such as the Atlanta Classification [56], Ranson’s criteria [57], or APACHE-II score [58] were used only in two RCTs [47,49]. Although CMR is advantageous in that it can be easily calculated after trial completion, clinical interpretation of case severity based on CMR requires prudent consideration, as it is subject to factors such as care quality provided at each institution. Unfortunately, accurate evaluations of the severity of acute pancreatitis are difficult at the entry of each trial. With reluctance, the present study used CMR as a surrogate measure of severity because only two trials [34,49] had used either the Ranson’s score or APACHE-II score.

The second limitation was the low quality of the individual studies evaluated by our review. The overall mean Jadad score was 2.1 (range, 0–5; three RCTs scored a 0 [28,32,43]), which meant that the overall quality of the studies was low to moderate. Our previous meta-analysis [10] showed that protease inhibitors were effective when CMR was greater than 0.10, while this was true only when CMR was greater than 0.20 in the present study. In our previous report [10], we calculated the APACHE-II scores using a multiple logistic equation described by Knaus et al. [58]. We also found that CMR = 0.10 scored roughly six points for the APACHE-II score method. On the APACHE-II scale (highest score: 67 points), we discovered that our patients scored 6 points when they presented with high-grade fever, hyperventilation, acidosis, renal dysfunction, leukocytosis, and deterioration of consciousness. One study has already validated the severity of acute pancreatitis with the APACHE-II score system [58]. CMR >0.20 for acute pancreatitis may be worse than these physical conditions, and we would be interested to test whether protease inhibitors would be effective in preventing death or other complications. Although meta-analyses of the two trials with CMR >0.20 showed that protease inhibitors were effective, reporting quality and heterogeneity should be taken into account when interpreting the results [34,49].

Third, the treatment modality of protease inhibitors was confined to intravenous administration. The present study excluded trials of intra-arterial or intra-abdominal infusion of protease inhibitors because the main objective was to update our previous meta-analysis [10]. Accordingly, we did not evaluate the effectiveness of protease inhibitors according to their various administrations. Further systematic reviews and meta-analyses on these modalities of administrations are expected in future. Future RCTs in this field should examine the effectiveness of protease inhibitors administered through intravenous or intra-arterial infusion, particularly among patients with severe acute pancreatitis.

## Conclusions

This updated meta-analysis re-confirmed that there is no solid evidence to support the use of intravenous protease inhibitors for preventing death, abdominal pain, pseudocyst formation, intra-abdominal abscess formation, surgical intervention, bowel obstruction, or any complications of pancreatitis excluding post-ERCP complications. Future trials on the effectiveness of intravenous protease inhibitor using established severity criteria (i.e., APACHE-II score system) should be promoted, particularly for patients with severe acute pancreatitis.

## Abbreviations

RCT: Randomized controlled trial; CMR: Control mortality rate; UMIN: University hospital medical information network; JMACCT: Japan medical association clinical trial registry; JAPIC: Japan pharmaceutical information center; RD: Risk difference; ERCP: Endoscopic retrograde cholangiopancreatography; GRADE: Grading of recommendations assessment, development and evaluation; M-H: Mantel-Haenszel; D-L: DerSimonian-Laird; NNT: Number needed to treat; NNTB: Number needed to treat benefit; NNTH: Number needed to treat harm; CI: Confidence Interval; ITT: Intention to treat; APCHE: Acute pathophysiology and chronic health evaluation; SE: Standard error.

## Competing interests

Potential competing interest: The authors declare that they have no competing interest.

## Authors’ contributions

TS contributed to the study design, literature search and selection, data extraction and analysis, and writing the draft. YN, SS, and TN contributed to the literature search and selections, data extraction and analysis. All authors read and approved the final manuscript.

## Pre-publication history

The pre-publication history for this paper can be accessed here:

http://www.biomedcentral.com/1471-230X/14/102/prepub

## Supplementary Material

Additional file 1: Table S1 Characteristics of primary trials. Click here for file

## Support

This study was supported by the grant-in-aid by the Ministry of Health, Labour and Welfare, Japan.
