# Supplementary figures and images for: Treatment of acute pancreatitis with protease inhibitors administered through intravenous infusion: an updated systematic review and meta-analysis
Source: BMC Gastroenterol. 2014 May 30;14:102. doi: 10.1186/1471-230X-14-102 (PMC4061927; doi:10.1186/1471-230X-14-102)

Table S1. Characteristics of primary trials.


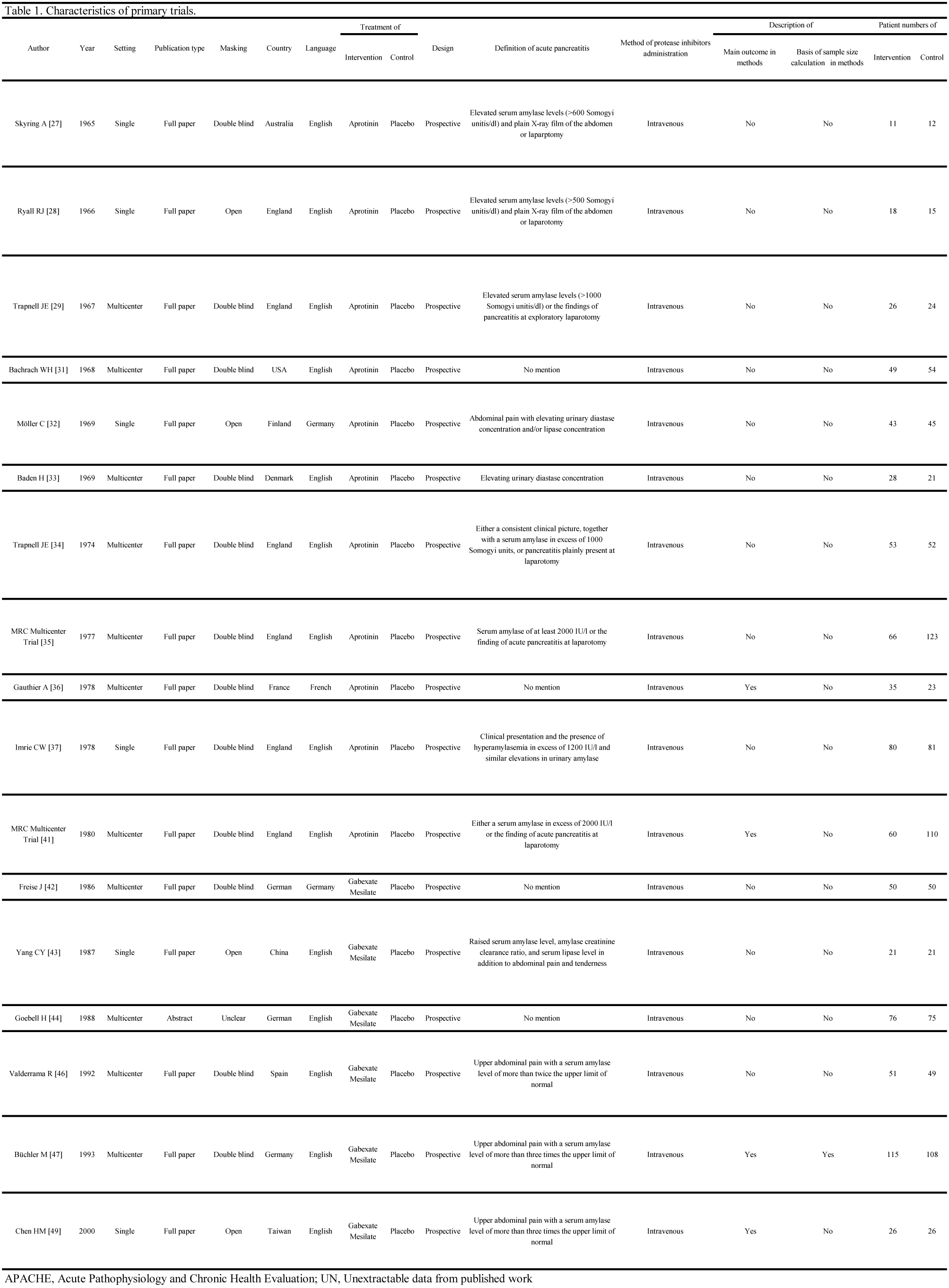


Table 1 cont’d .


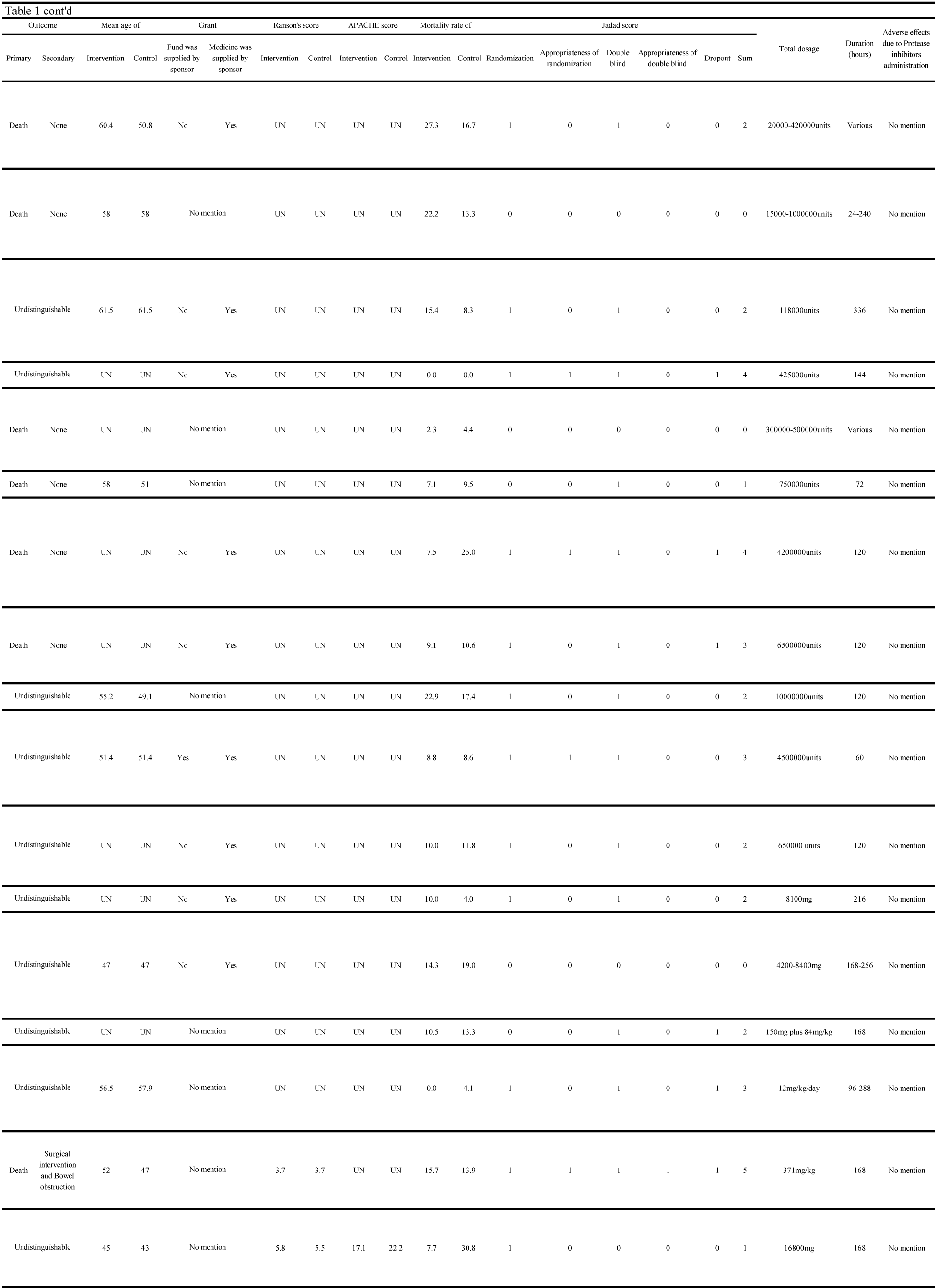

Supplement: Additional file 1: Table S1 — Characteristics of primary trials. [file 1471-230X-14-102-S1.doc]
